# Metabolic Syndrome in South African Patients with Severe Mental Illness: Prevalence and Associated Risk Factors

**DOI:** 10.1371/journal.pone.0149209

**Published:** 2016-02-16

**Authors:** Shamima Saloojee, Jonathan K Burns, Ayesha A Motala

**Affiliations:** 1 Department of Psychiatry, Nelson R Mandela School of Medicine, University of KwaZulu - Natal, Durban, South Africa; 2 Department of Diabetes and Endocrinology, Nelson R Mandela School of Medicine, University of KwaZulu - Natal, Durban, South Africa; Maastricht University, NETHERLANDS

## Abstract

**Background:**

There is a surge of cardiovascular disease (CVD) in Africa. CVD is the leading cause of mortality among patients with severe mental illness (SMI) in developed countries, with little evidence from the African context.

**Objective:**

To determine the prevalence and risk factors for MetS among South African patients with SMI.

**Method:**

In a cross sectional study, individuals with SMI treated with antipsychotics and a control group without a mental illness, matched for age, gender and ethnicity were evaluated for MetS using the 2009 Joint Interim statement (JIS) criteria.

**Results:**

Of the 276 study group subjects, 65.9% were male, 84.1% black African, 9.1% white, 5.4% of Indian descent and 1.5% coloured (mixed race) with a mean age of 34.7 years (±12.5). Schizophrenia was the most common diagnosis (73.2%) and 40% were taking first generation antipsychotics. The prevalence of MetS was 23.2% (M: 15.4%, F: 38.3%) in the study group and 19.9% (M: 11.9%, F: 36.3%) in the control group (p = 0.4). MetS prevalence was significantly higher in study subjects over 55 years compared to controls (p = 0.03). Increased waist circumference (p< 0.001) and low high density lipoprotein (HDL) cholesterol (p = 0.003) were significantly more prevalent in study subjects compared to controls. In study subjects, risk factors associated with MetS included age (OR: 1.09, 95% CI 1.06–1.12, p < 0.001), female gender (OR: 2.19, 95% CI 1.06–4.55, p = 0.035) and Indian descent (OR: 5.84, 95% CI 1.66–20.52, p = 0.006) but not class of antipsychotic (p = 0.26).

**Conclusion:**

The overall MetS prevalence was not increased in patients with SMI compared to controls; however, the higher prevalence of the individual components (HDL cholesterol and waist circumference) suggests an increased risk for CVD, especially in patients over 55 years.

## Introduction

Metabolic syndrome (MetS) gained prominence in the psychiatric literature in the past decade because the leading cause of premature mortality in patients with severe mental illness (SMI) from developed countries is reported to be cardiovascular disease (CVD) [1–3]. There is little doubt that MetS is a risk factor for diabetes mellitus and CVD [4], although the clinical utility and criteria by which MetS is defined is debatable [5,6]. Regarding the elevated risk of CVD in SMI, there have been several reports on the prevalence and associated risk factors for MetS in SMI globally [7–12]. Reported prevalence rates range from 9.3% in Indonesia [8] to 21% in Mexico [9], 27.5% in Japan [10], 57% in England [11] and 68% in Australia [12]. In a recent meta—analysis on data from 27 countries for the period 2003–2011, Mitchell et al. reported an overall prevalence of 32.5% in patients with schizophrenia and related disorders [13].

There is limited information regarding the prevalence of MetS in patients with SMI from Africa. The meta—analysis by Mitchell et al.[13] did not include any studies from Africa; also, only 0.001% of the patients enrolled in schizophrenia trials throughout the world are recruited from Africa [14]. Data for South Africa are limited to three studies [15–17]. A study of 84 long term in patients that included patients with cognitive and personality disorders reported a MetS prevalence of 32% [15] while the other two reported on the prevalence of MetS in patients taking a single antipsychotic [16,17]. From population studies in South Africa, the reported prevalence of MetS is 22.1% in rural [18] and 31.7% in urban black South African communities [19].

In many high income countries, more than 80% of antipsychotic prescriptions are for second generation antipsychotics (SGAs) and the metabolic side effects and magnitude of risk attributable to SGA medication is well established [20–22]. However, evidence comparing the metabolic side effects of individual older and cheaper, first generation antipsychotics (FGAs) which are still widely prescribed in Africa [23] is mostly derived from studies conducted prior to 1994, and is of low quality [24] prompting a call for more recent studies [24,25].

Regarding risk factors for MetS in SMI, reports on the gender distribution of MetS in SMI is variable, because although many studies report a higher prevalence in females [7,9,26,27] some report a higher prevalence in males [10,12,28]. More than two thirds of the studies in the meta—analysis by Mitchell et al. [13] did not report on ethnicity, and there is limited and mixed evidence for the increased prevalence of MetS among ethnic minorities with SMI from the United States (US) [29].

The sparse information regarding the prevalence and risk factors for MetS in South African patients with SMI is of concern, because Mensah et al. have shown that from 1990 to 2013 there was an 81% increase in CVD deaths in Sub Saharan Africa (SSA), with more women dying of CVDs (512 269) than men (445 445) [30].

This study was therefore undertaken to determine the prevalence of MetS and associated risk factors in South African patients with SMI treated with antipsychotic medication.

## Methods

We conducted a cross—sectional study in the psychiatric unit at King Edward VIII Hospital (KEH) in Durban, South Africa from February 2012 to December 2014. KEH is a 922 bedded general hospital with 20 psychiatric beds and serves a population of approximately 360 000 individuals. The psychiatric unit has two full time psychiatrists and provides a regional level service for other district hospitals without psychiatrists; but also serves as a district hospital because it is close to a large informal settlement without community mental health clinics. The study subjects were 18–65 year old patients with SMI of black, white, Asian Indian or coloured (mixed) ethnicity. In—or out—patients with schizophrenia, bipolar 1 mood disorder or schizoaffective disorder diagnosed using the Diagnostic and Statistical Manual of Mental Disorders (4th ed., text rev.; DSM-IV-TR; American Psychiatric Association, 2000) [31] criteria on antipsychotic medication for at least three months were included. Subjects who were taking a single antipsychotic only were assigned to the monotherapy group. Those subjects who were on two or more antipsychotics at the same time for at least six weeks and those who were taking antipsychotics combined with sodium valproate or an antidepressant were assigned to the polytherapy group. Control subjects were recruited by placing posters explaining the purpose and procedures for the study at the entrance, exit and other high traffic areas in the hospital. Hospital staff, health science students and members of the public who were physically healthy and who had no lifetime diagnosis or treatment for a mental illness were invited to participate in the study. Control and study subjects who were HIV positive or pregnant were excluded. Control subjects were matched for age gender and ethnicity with study subjects.

All consenting study and control subjects were interviewed and information regarding demographic and clinical characteristics was recorded on a specifically designed questionnaire. Study subject records were examined for diagnosis of mental illness and currently administered antipsychotic. At KEH, chlorpromazine and haloperidol are first line antipsychotic agents with risperidone and clozapine as alternatives. Olanzapine, quetiapine, aripiprazole and amisulpride are available only by special motivation.

### Ethics Statement

This study was approved by the Biomedical Research Ethics Committee of the University of KwaZulu—Natal. Written informed consent was obtained from all study and control subjects in English or Isizulu.

Anthropometric measurements were conducted as per the World Health Organization (WHO) (2000, 1995) protocols [32]. Weight in kilograms (kg) and height in centimetres (cm) were measured for calculation of the body mass index (BMI). BMI (kg/m^2^) was categorized as normal (< 25), overweight (25–29.9) and obese (> 30). Waist circumference was measured using a soft tape measure at the mid -point between the upper border of the iliac crest and the inferior margin of the last rib. Blood pressure was measured with the subject in the sitting position after a ten minute rest; two readings were taken with a minimum interval of ten minutes and the mean of the two readings was used to record the blood pressure. Venous blood sampling was performed after an overnight fast for plasma glucose and serum lipids (total cholesterol, total triglycerides, high density lipoprotein [HDL] cholesterol, low density lipoprotein [LDL] cholesterol). Plasma glucose, HDL and triglycerides were measured by the enzymatic method utilizing Beckman Coulter GLU, HDLD and TG reagent respectively on a Beckman Coulter DxC 600 instrument. LDL cholesterol was calculated by the Freidewald formula.

MetS was defined using the 2009 Joint Interim Statement (JIS) definition [4] and is diagnosed by the presence of any 3 of the following 5 risk factors: (i) increased waist circumference; waist circumference cut off points used: whites, blacks and coloureds: men ≥ 94 cm, women ≥ 80 cm; Indians: men ≥ 90 cm, women ≥ 80 cm (ii) elevated blood pressure: systolic >130 mmHg (or on treatment) and/or diastolic >85 mmHg (or on treatment) (iii) elevated fasting plasma glucose ≥ 5.6 mmol/l (or on treatment) (iv) elevated fasting triglycerides ≥1.7 mmol/l (or on treatment) and (v) reduced HDL cholesterol ≤1.0 mmol/l men (or on treatment), ≤ 1.3 mmol/l women (or on treatment).

### Statistical analysis

All statistical analyses were performed using SAS version 9.3 (SAS Institute Inc,. Cary).

The demographic and clinical characteristics of participants were summarized using means ± standard deviation (SD) or medians and interquartile ranges (IQR) for continuous variables and as proportions (%) for categorical variables. To compare differences in continuous demographic and clinical variables between the study and control groups and by class of antipsychotic medication, Student’s t-test, F-test, Wilcoxon-Mann-Whitney test and Kruskal-Wallis test were used depending on the data distribution. Fisher’s exact test was used to compare categorical variables. The difference in the prevalence of MetS and it’s individual components between the study and control groups and by class of antipsychotic medication was determined using Fisher’s exact test. To determine risk factors associated with MetS, multivariate logistic regression models were used were used. Odds ratios (OR) and 95% confidence intervals (95% CI) were calculated to determine the independent risk factors associated with MetS. A *p* value <0.05 was deemed to be statistically significant.

## Results

### Study group and control group

Table 1 shows the clinical and laboratory characteristics of the study and control subjects. There were 276 subjects with a SMI in the study group (M:F 182: 94) and majority were of black African ancestry (84.1%). There were no significant differences in the demographic characteristics between the two groups. In the study group, the majority (73.2%) had a diagnosis of schizophrenia with a mean duration of illness of 4.6 (±5.21) years. The most commonly prescribed antipsychotic alone or in combination with other antipsychotics was risperidone (33.9%), and the least commonly prescribed was olanzapine (3.67%). The mean BMI and waist circumference were high in both the study (27.6 ± 7.4 kg/m^2^; 92.8 ± 15cm, respectively) and control (27.1 ± 7.4 kg/m^2^; 87.8 ± 15.5cm, respectively) groups. Over half of the study group (55%) and control group (50%) were either overweight or obese.

**Table 1:** Clinical and laboratory characteristics of patients with severe mental illness (study group) and control group.

	Study group (n = 276)	Control group (n = 276)	p
Gender			0.86 ^a^
Male	65.9(182)	67.0(185)	
Female	34.1 (94)	33.0(91)	
Age(years)	34.7 ± 12.5	34.7 ± 12	0.94 ^b^
Age group(years)(% [n])			
18–24	25.72(71)	25.36(70)	
25–34	34.06(94)	34.78(96)	
35–44	17.03(47)	16.67(46)	
45–54	13.04(36)	13.41(37)	
≥ 55	10.15(28)	9.78(27)	
Ethnicity			1.00 ^a^
African	84.1(232)	84.1(232)	
White	9.1(25)	9.1(25)	
Indian	5.4(15)	5.8(16)	
Coloured(mixed race)	1.5(4)	1.1(3)	
Diagnosis			
Schizophrenia	73.2(202)		
Schizoaffective disorder	15.9(44)		
Bipolar mood disorder	10.9(30)		
First generation antipsychotic medication			
Zuclopenthixol Decanoate	14.12(50)		
Chlorpromazine	10.17(36)		
Flupenthixol Decanoate	7.91(28)		
Haloperidol	7.91(28)		
Second generation antipsychotic medication			
Risperidone	33.90(120)		
Clozapine	8.19(29)		
Amisulpride	5.93(21)		
Aripiprazole	4.24(15)		
Quetiapine	3.96(14)		
Olanzapine	3.67(13)		
Cigarette smokers	41.3(114)	34.8(96)	0.14 ^a^
Body Mass Index (BMI) (kg/m^2^)	27.6 ±7.4	27.1 ± 7.4	0.46 ^b^
BMI category:			0.46 ^a^
Normal: < 25	45.3(125/276)	50.2(138/275)	
Overweight: 25–29.9	26.5(73/276)	22.6(62/275)	
Obese: > 30	28.3(78/276)	27.3(75/275)	
Waist circumference (cm)	92.8 ± 15.0	87.8 ± 15.5	<0.001 ^b^
Blood pressure(mmHg):			
Systolic	121.9 ± 16.2	121.8 ± 17.9	0.95 ^b^
Diastolic	76.2 ± 10.2	76.9 ± 12.6	0.45 ^b^
Fasting plasma glucose (mmol/l)	5.1 ± 1.2	5.1 ± 1.3	0.99 ^b^
Serum lipids (mmol/l):			
Total cholesterol	4.1 ± 1.1	4.1 ± 1.0	0.96 ^b^
Total triglycerides	1.1 ± 0.7	1.0 ± 0.7	0.40 ^b^
High density lipoprotein cholesterol	1.1 ± 0.3	1.2 ± 0.3	0.01 ^b^
Low density lipoprotein cholesterol	2.5 ± 0.9	2.5 ± 0.8	0.80 ^b^

Data are mean ± SD and % (n), unless otherwise indicated p for significant differences between study and control group analysed using

^a^ Fisher’s exact test and,

^b^ Student’s t—test.

### Prevalence of MetS and individual components

The overall prevalence of MetS was 23.2% in the study group and 19.9% in the control group with no significant difference between the two groups (p = 0.4). In both groups the prevalence was higher in women. The prevalence of MetS in the oldest age group was significantly higher in the study group (71.4%) compared to the control group (40.7%) (p = 0.03). In the study group, subjects of Indian descent (60%) and those with schizoaffective disorder (36.4%) had a higher prevalence of MetS compared to African subjects (19.4%) and those with schizophrenia (20.8%) respectively (Table 2). Compared to men, women with schizophrenia (36.4% vs 15%, p = 0.001) and schizoaffective disorder (61.1% vs 19.2%, p = 0.005), but not bipolar disorder (23.8% vs 11.1%, p = 0.4) had a significantly higher prevalence of MetS.

**Table 2:** Prevalence of metabolic syndrome by demographic characteristics and diagnosis in the study and control groups.

	Study group (n = 276)	Control group (n = 276)	p ^a^
Total	23.2(64)	19.9(55)	0.41
Gender			
Male	15.4(28/182)	11.9(22/185)	0.36
Female	38.3(36/94)	36.3(33/91)	0.88
Age Group (years)			
18–24	11.3(8/71)	5.7(4/70)	0.37
25–34	7.5(7/94)	9.4(9/96)	0.80
35–44	27.7(13/47)	26.1(12/46)	1.00
45–54	44.4(16/36)	51.4(19/37)	0.64
≥55	71.4(20/28)	40.7(11/27)	0.03
Ethnicity			
African	19.4(45/232)	17.7(41/232)	0.72
White	36.0(9/25)	32.0(8/25)	1.00
Indian	60.0(9/15)	37.5(6/16)	0.29
Coloured (mixed race)	25.0(1/4)	0(0/3)	
Diagnosis			
Schizophrenia	20.8(42/202)		
Schizoaffective disorder	36.4(16/44)		
Bipolar mood disorder	20.0(6/30)		

Data are % (n).

^a^ Fisher’s exact test.

For the individual components of MetS (Table 3), the most frequent abnormality in both the study and control groups was increased waist circumference (55.1% and 40.6%, study and control groups, respectively); the least frequent component was elevated serum triglycerides (14.5% and 12.3%, respectively). The prevalence of low serum HDL cholesterol was significantly higher in the study group (52.5%) than the control group (39.9%) (p = 0.003).

**Table 3:** Prevalence of the individual components of metabolic syndrome in the study and control groups.

	Study group (n = 276)	Control group (n = 276)	OR (95%CI)	p
Metabolic Syndrome (MetS)	23.2(64)	19.9(55)	1.21(0.81–1.82)	0.35 ^a^
Individual components:				
Increased waist circumference (cm)	55.1(152)	40.6(112)	1.79(1.28–2.52)	<0.001 ^a^
Elevated blood pressure (mmHg):				
Systolic	17(47)	24.3(67)	0.64(0.42–0.97)	0.05 ^a^
Diastolic	23.2(64)	26.8(74)	0.82(0.56–1.21)	0.38 ^a^
Elevated fasting plasma glucose (mmol/)	17.4(48)	13.8(38)	1.32(0.83–2.09)	0.24 ^a^
Elevated serum triglycerides (mmol/l)	14.5(40)	12.3(34)	1.21(0.74–1.97)	0.45 ^a^
Low HDL –cholesterol (mmol/l)	52.5(145)	39.9(110)	1.67(1.19–2.34)	0.003^a^
Number of MetS criteria met:				0.06 ^b^
0	19.6(54)	30.8(85)		
1	27.5(76)	26.8(74)		
2	29.7(82)	22.5(62)		
3	15.9(44)	13.8(38)		
4	4.4(12)	4.0(11)		
5	2.9(8)	2.2(6)		

Data are % (n). HDL: high density lipoprotein.

^a^ z test.

^b^ Fisher’s exact test.

### Prevalence of MetS and antipsychotic medication

All study subjects were on antipsychotic medication (n = 276), with127 subjects (46%) on monotherapy and 149 (54%) on polytherapy. Of the 149 subjects on polytherapy, 98 (35.5%) were taking an antipsychotic combined with sodium valproate, and 51 (15.5%) were taking two or more antipsychotics at the same time.Of the 98 subjects on sodium valproate 6 (2.2%) subjects were also taking an antidepressant (5 subjects were on a Selective Serotonin Reuptake Inhibitor and 1 was on a Serotonin Noradrenalin Reuptake Inhibitor) and only one subject was taking lithium.

There was no significant difference (p = 0.9) in the prevalence of MetS in subjects on antipsychotic polytherapy (35/149, 23.5%) compared to those on monotherapy (29/127, 22.8%). In addition, no difference (p = 0.8) was observed in the prevalence of MetS in subjects on antipsychotic monotherapy (22.8%) compared to subjects taking antipsychotics combined with sodium valproate and antidepressant medication (24.5%) or those subjects taking two or more antipsychotics (21.6%, p = 0.9). Too few subjects were taking antipsychotics combined with antidepressants and sodium valproate only for a separate analysis.

Regarding the comparison of metabolic abnormalities in subjects taking FGAs vs SGAs; of the 127 subjects on monotherapy, 32 (11.6%) subjects were taking FGAs and 95 (34.4%) SGAs. No significant difference was observed in the clinical and demographic characteristics between subjects taking FGAs or SGAs as monotherapy (Table 4). There was no difference in the prevalence of MetS or its individual components between subjects on FGA vs SGA monotherapy (p = 0.26) (Table 5) or polytherapy (p = 0.2) (data for polytherapy not shown).

**Table 4:** Demographic and clinical characteristics of subjects with severe mental illness by class of antipsychotic medication in subjects on monotherapy.

	Subjects taking FGAs (n = 32)	Subjects taking SGAs (n = 95)	p
Gender			0.7 ^a^
Male	65.6(21)	69.5(66)	
Female	34.4(11)	30.5(29)	
Age (years)	33.7 ±13.8	33.3 ± 12.3	0.9 ^b^
Diagnosis			0.5 ^a^
Schizophrenia	96.9(31)	88.4(84)	
Schizoaffective disorder	3.1 (1)	9.5 (9)	
Bipolar mood disorder	0(0)	2.1 (2)	
Body Mass Index (BMI) (kg/m^2^)	26.6 ± 7.57	27.5 ± 6.31	0.5 ^b^
BMI category:			0.5 ^a^
Normal: < 25	59.3(19)	47.4(45)	
Overweight: 25–29.9	18.8(6)	23.2(22)	
Obese: >30	21.9(7)	29.5(28)	
Waist circumference (cm)	90.5 ± 15.4	92.3 ± 16	0.6 ^b^
Blood pressure (mmHg):			
Systolic	119.2 ± 11.3	122.5 ±17.9	0.3 ^b^
Diastolic	72.6 ± 8.5	76.6 ± 10.2	0.04 ^b^
Fasting plasma glucose (mmol/l)	5.2 ± 1.3	5.3 ± 1.4	0.7 ^b^
Serum Lipids (mmol/l):			
Total cholesterol	4.2 ± 1.1	4.2 ±1.1	0.7 ^b^
Total triglycerides	0.9 ± 0.4	1.1 ± 0.7	0.08 ^b^
High Density Lipoprotein cholesterol	1.2 ± 0.3	1.1 ± 0.3	0.2 ^b^
Low Density Lipoprotein cholesterol	2.6 ± 1.0	2.6 ±0.9	0.7 ^b^

Data are mean ± SD and % (n), unless otherwise indicated. FGA: first generation antipsychotic, SGA: second generation antipsychotic.

^a^ Fisher’s exact test,

^b^ Student’s t—test.

**Table 5:** The prevalence of metabolic syndrome and individual components according to class of medication in subjects on antipsychotic monotherapy.

	FGA (n = 32)	SGA (n = 95)	p ^a^
Metabolic Syndrome	15.6(5)	25.3(24)	0.26
Elevated waist circumference (cm)	62.5(20)	51.6(49)	0.28
Elevated blood pressure (mmHg):			
Systolic	6.25(2)	20(19)	0.07
Diastolic	6.25(2)	21.1(20)	0.06
Elevated fasting plasma glucose (mmol/l)	18.8(6)	25.3(24)	0.45
Elevated serum triglycerides (mmol/l)	3.1(1)	6.3(6)	0.5
Low HDL –cholesterol (mmol/l)	43.8(14)	52.6(50)	0.38

Data are % (n). HDL: high density lipoprotein. FGA: first generation antipsychotic, SGA: second generation antipsychotic.

^a^ z test.

### Risk factors associated with MetS

In multivariate analysis, significant risk factors associated with MetS included age (OR: 1.09, 95% CI 1.06–1.12, p <0.0001), female gender (OR: 2.19, 95% CI 1.06–4.55, p = 0.035) and Indian descent (OR: 5.84, 95% CI 1.66–20.52, p = 0.006) (Table 6).

**Table 6:** Multivariate analysis of risk factors associated with metabolic syndrome in the study and control groups.

	Study group		Control group	
	*Odds Ratio (95% CI)	p ^a^	*Odds Ratio (95% CI)	p ^a^
Female gender	2.19(1.06–4.55)	0.04	1.31(0.57–3.00)	0.53
Age	1.09(1.06–1.12)	<0.001	1.07(1.03–1.10)	<0.001
Ethnicity:				
Indian	5.84(1.66–20.52)	0.006	3.80(1.12–12.89)	0.03
White and mixed race	0.51(0.03–8.77)	0.64	1.68(0.55–5.13)	0.36
Body Mass Index (kg/m^2^)	1.13(1.07–1.19)	<0.001	1.16(1.09–1.23)	<0.001

*adjusted values.

^a^ *chi*-squared test

## Discussion

In this study of South African patients with SMI taking antipsychotic medication there was a high but similar prevalence of MetS in the study (23.2%) and control group (19.9%); the prevalence of the individual components viz. increased waist circumference (p< 0.001) and low HDL cholesterol (p = 0.003) was higher in the study group. Significant risk factors for MetS in the study group included age, gender and Indian ethnicity. No significant difference (p = 0.9) was observed in the prevalence of MetS in subjects on monotherapy compared to those on polytherapy and in subjects taking FGAs or SGAs as monotherapy (p = 0.26).

The prevalence in this study is in keeping with rates reported from Thailand (22.8%) [33], Mexico (21%) [9] and Spain (26.5%) [34], but lower than studies from England (57%) [11], the US (49.2%) [26], Australia (68%) [12], and the overall international prevalence of 32.5% reported by Mitchell et al.[13]. A review by Papanastasiou on the prevalence of MetS in schizophrenia [22], the position statement by De Hert [1] and meta –analysis by Mitchell et al. [13] found that many studies reported a two—fold higher prevalence of MetS in patients with SMI compared to the general population. However, no difference in the prevalence between SMI and control subjects was reported from Venezuela [35] and Mexico [9]. There are several possible explanations for the lower prevalence of MetS in the study group participants including the high proportion of young participants (25.6% of the study subjects were < 25 years), male participants (66% male), the short mean duration of illness (4.6 years), the inclusion of participants with a first episode of SMI and the low proportions of participants taking FGA and SGA medication (chlorpromazine 10.2%, clozapine 8.2% and olanzapine 3.7%) that are associated with the highest risk for MetS [36].

The high prevalence of increased waist circumference in subjects with SMI in this study is compatible with findings of other studies [1,13,21,22]. In a recent report, more than 80% of patients taking antipsychotic medication had central obesity [11]. This places patients with SMI at increased risk for diabetes mellitus, given that central obesity correlates better with insulin resistance than total body obesity (BMI) [37]. The prevalence of diabetes mellitus is reported to be approximately 12% in patients with SMI and two to three fold higher than that in the general population [38]. However, we did not find a high prevalence of dysglycemia in patients with SMI confirming an earlier study from this district which reported a low prevalence (3.85%) of type 2 diabetes mellitus in a group of chronic hospitalized patients with SMI [39]. Although genetic and lifestyle factors may also account for abnormal glucose homeostasis and frank diabetes with antipsychotic treatment, it is likely that the low prevalence of diabetes mellitus in this study is due to the underutilization of antipsychotics with a higher liability for diabetes mellitus such as clozapine, olanzapine and quetiapine [40]. Prospective studies in African patients with SMI are required to further clarify the risk of dysglycemia.

In this study, 18,5% of patients were prescribed two or more antipsychotics, and 35,5% were on antipsychotics combined with sodium valproate. There is little empirical support for either practice but both are common internationally [41] with rates of 2–70% [42] and 16–35% [43] respectively. Although it has been shown that combining psychotropic medications results in the potentiating of the metabolic side effects of each of the individual medications [44], the results of this study support and extend the evidence for no independent association between MetS and the prescription of two or more antipsychotics [45] or antipsychotics combined with sodium valproate [46].

Furthermore, an additional analysis showed that there was no difference in the prevalence of MetS between subjects taking FGAs and SGAs as monotherapy (p = 0.26) or polytherapy (0.2). As reported in the literature, possible explanations include the probability that there is no class effect for the metabolic adverse effects of antipsychotics in view of the substantial heterogeneity within each class [21,25,47]; and the current classification of antipsychotics is arbitrary [21]. Taking into account that subjects in this naturalistic study were not randomized to either class of medication and that the comparison by class of antipsychotic medication is a within group analysis, it is also likely that the sample size is insufficient to detect a difference between FGAs and SGAs. However, describing the metabolic side effects of FGAs is still relevant because although many SGA medications are now off patent, two of the three oral antipsychotics on the 19th WHO Model List of Essential Medicines (April 2015) are FGAs [48].

In a multivariate analysis, significant risk factors associated with MetS were age, female gender and Indian descent. The association of age with MetS that we observed in subjects and controls concurs with that reported in the literature [13,22,28]. However, the higher prevalence of MetS in subjects with SMI under 25 years (11.3%) compared to controls (5.7%) is of concern and consistent with recent reports of the development of MetS in young patients with SMI [49].

The significant association of female gender with MetS in this study confirms the findings of the landmark CATIE study, one of the largest antipsychotic effectiveness studies to date [26], by contrast, the meta-analysis by Mitchell et al. [13] reported no significant gender difference, while a Korean study found that the risk of MetS was four fold higher in males with schizophrenia[28]. The increased risk for MetS in women compared to men that we observed in the study group may reflect a regional predisposition to obesity and MetS in women because a higher prevalence of obesity and MetS in South African women has been confirmed in epidemiological studies [18,19]. Culturally acceptable attitudes to larger body size may be influencing this phenomenon [50]. Our findings may also be explained by an increased susceptibility to the side effects of antipsychotic medication in women with SMI [51],because in contrast to all the other risk factors for MetS in this study, the increased risk for MetS in women compared to men in the control group, was not statistically significant (p = 0.53). However, even though South African women have a higher prevalence of individual risk factors for CVD such as obesity, diabetes mellitus and HDL cholesterol, a recent study has shown a higher 10 year Framingham risk for CVD in men [52].

Racial differences in the risk for MetS has been documented in patients with SMI previously [29]. However, the elevated risk for MetS that we observed in South Africans of Indian descent with SMI compared to Africans with SMI has to the best of our knowledge not been documented previously. A likely explanation for the high prevalence of MetS in native and migrant Indians include a genetic predisposition to diabetes mellitus and a tendency for higher visceral fat deposition at lower BMI’s than other ethnic groups [53]. We did not confirm the findings of Ader et al [54] who found that African Americans were more susceptible to the metabolic side effects of antipsychotic medication but our findings were in keeping with those of Keenan et al [55] who found a lower risk for MetS in African American subjects with SMI after adjusting for age and gender. This substantiates the need for further studies on the association of ethnicity and MetS among those with SMI in and outside the US.

The limitations of this study include the cross sectional, observational study design that resulted in disproportionate medication groups, lack of information on lifestyle factors and the recruitment of participants from a single site which limits the generalizability of the results.

## Conclusion

The results of this study show that 1 in 5 African patients with SMI taking either FGA or SGA medication met the criteria for MetS, and 1 in 2 was overweight or obese. Moreover, the development of MetS in females, individuals of Indian descent and young patients under the age of 25 years on treatment with antipsychotics is of concern. Our results emphasise the need for nationwide, cardiovascular and metabolic risk preventative and screening programmes that must include individuals with a mental illness. This study provides evidence for the integration of physical health programmes into mental health services in South Africa.
